# Intrarater and Interrater Reliability of the Flexicurve Index, Flexicurve Angle, and Manual Inclinometer for the Measurement of Thoracic Kyphosis

**DOI:** 10.1155/2013/475870

**Published:** 2013-12-12

**Authors:** Eva Barrett, Karen McCreesh, Jeremy Lewis

**Affiliations:** ^1^University of Limerick, Limerick, Ireland; ^2^Department of Allied Health Professions and Midwifery, School of Health and Social Work, Wright Building, College Lane Campus, University of Hertfordshire, Hatfield, Hertfordshire AL10 9AB, UK; ^3^Musculoskeletal Services, Health at the Stowe, Central London Community Healthcare NHS Trust, 260 Harrow Road, London W2 5ES, UK

## Abstract

*Objective*. This study aimed to describe the interrater and intrarater reliability of the flexicurve index, flexicurve angle, and manual inclinometer in swimmers. A secondary objective was to determine the level of agreement between the inclinometer angle and the flexicurve angle and to provide an equation to approximate one angle from the other. *Methods*. Thirty swimmers participated. Thoracic kyphosis was measured using the flexicurve and the manual inclinometer. Intraclass correlation coefficient, 95% confidence interval, and standard error of measurement were computed. *Results*. The flexicurve angle and index showed excellent intrarater (ICC = 0.94) and good interrater (ICC = 0.86) reliability. The inclinometer demonstrated excellent intrarater (ICC = 0.92) and interrater (ICC = 0.90) reliability. The flexicurve angle was systematically smaller and correlated poorly with the inclinometer angle (*R* ^2^ = 0.384). The following equations can be used for approximate conversions: flexicurve angle = (0.275 × inclinometer angle) + 8.478; inclinometer angle = (1.396 × flexicurve angle) + 8.694. *Conclusion*. The inclinometer and flexicurve are both reliable instruments for thoracic kyphosis measurement in swimmers. Although the flexicurve and inclinometer angles are not directly comparable, the approximate conversion factors provided will permit translation of flexicurve angle to inclinometer angle and vice versa.

## 1. Introduction

Thoracic kyphosis is the sagittal plane curvature between the T1 and T12 vertebral bodies [1]. Normal thoracic kyphosis ranges from 20° to 50° when assessed radiographically [2] and nonradiographically [2–4]. Excessive thoracic kyphosis, defined as a kyphosis > 50° [2, 5], has been linked with a range of musculoskeletal complaints including shoulder pain [6] and cervical pain [7–9].

Previous research has consistently reported high incidences of shoulder pain in competitive swimmers, with rates of 53% [10], 54% [11], and 80% [12] amongst those documented. The increased thoracic kyphosis of swimmers is a postural adaptation to altered spinal forces experienced in swim training [13] and is proposed as being a large contributing factor to the development of shoulder pain [14, 15]. The simple and safe assessment of thoracic kyphosis is therefore of value to physiotherapists involved in treating high-level swimmers.

The gold standard for the measurement of thoracic kyphosis is a radiograph, which provides a Cobb angle [16, 17]. While this method is noted to reveal the true position of the vertebrae [18], it is not always accessible in a clinical setting, involves high costs, and exposes the patient to potentially harmful radiation [19, 20]. Consequently, a wide range of noninvasive instruments have been developed for the clinical measurement of thoracic kyphosis. These methods include the arcometer [18, 21], 3D ultrasound [22], Debrunner's kyphometer [19, 23], Spinal Mouse [3, 20], photogrammetry [4, 24], goniometry [25], and electrogoniometry [1]. Currently, there is no conclusive evidence regarding which of these tools is the most reliable or valid. The flexicurve and manual inclinometer are two hand-held tools which are commonly used by physiotherapists for the measurement of thoracic kyphosis. These are simple, quick, and cost effective, making them suitable to use at poolside.

The primary output of the flexicurve is the kyphosis index. Previously, the flexicurve index has shown very high interrater and intrarater reliability in a healthy population [5, 26] and an osteoporotic population [23, 27]. However, the reliability of the flexicurve index in swimmers has not yet been investigated. The flexicurve does not provide an immediate angle which limits its clinical interpretation. Recently, Greendale et al. [23] introduced a geometric formula to translate the flexicurve index into an approximate Cobb angle. As access to radiological assessment is sometimes limited, it would be of value to compare the flexicurve angle to another commonly used clinical tool like the inclinometer. Similar to the flexicurve index, very high intrarater reliability has been reported for the manual inclinometer in nonathletic subjects [28, 29]. To the knowledge of the investigators, the inter-rater reliability of the manual inclinometer has not yet been reported in the literature in any population, which is a significant gap in the literature.

The primary purpose of this study was to investigate the intrarater and interrater reliability of the manual inclinometer, flexicurve index, and flexicurve angle in a population of swimmers. The secondary purpose was to compare the inclinometer angle and the flexicurve angle obtained using the method presented previously [23].

## 2. Methods

### 2.1. Subjects

For a significance level of 5% and a power of 80, the suggested adequate number of subjects required is 19 [30, 31]. Thirty subjects (18 male, 12 female) participated in this study. This is an equal sample size to that used in a previous similar reliability study [29]. Subjects were recruited by email to local swimming clubs. Inclusion criteria were as follows: member of a swimming club, swimming at least twice per week, and aging at least 18 years. Subjects with and without shoulder pain were accepted for participation. Clinical tests were not used to identify the source of shoulder pain due to the low level of validity and reliability of diagnostic shoulder tests [32, 33]. Thus this study examined individuals with nonspecific shoulder pain. Permission to conduct this study was granted by the University of Limerick Ethics Committee. All subjects signed a witnessed informed consent form and were aware of their rights including the right to withdraw from the study at any stage. Each participant completed a questionnaire detailing swim training and shoulder pain history.

### 2.2. Raters

To evaluate interrater and intrarater reliability of the instruments, two raters were used. Both raters were qualified physiotherapists, with- one and 15-year experience, respectively, in the use of the instruments for clinical and research purposes. The raters also had a practice session with both instruments prior to commencement of the study. This included a review of basic spine anatomy, instruction in how to find landmarks by palpation and practice with the use of both instruments, and how to take readings from them. This ensured a good level of familiarity with both techniques.

### 2.3. Procedure

This study was conducted alongside other investigations which assessed other components of upper body posture and strength. At first, the subject was asked to lie prone and the spinous processes of C7, T1, T2, T12, and L1 were identified by palpation and marked with an easily removable marker. The interspinous space of L3/4 was identified at the level of the iliac crests and the L1 and T12 spinous processes were marked by palpating superiorly from this reference point [34]. The 7th cervical vertebra was designated to have the most prominent spinous process [34]. Palpating inferiorly from this reference point, the T1 and T2 spinous processes were identified and marked [28].

**Figure 1:**
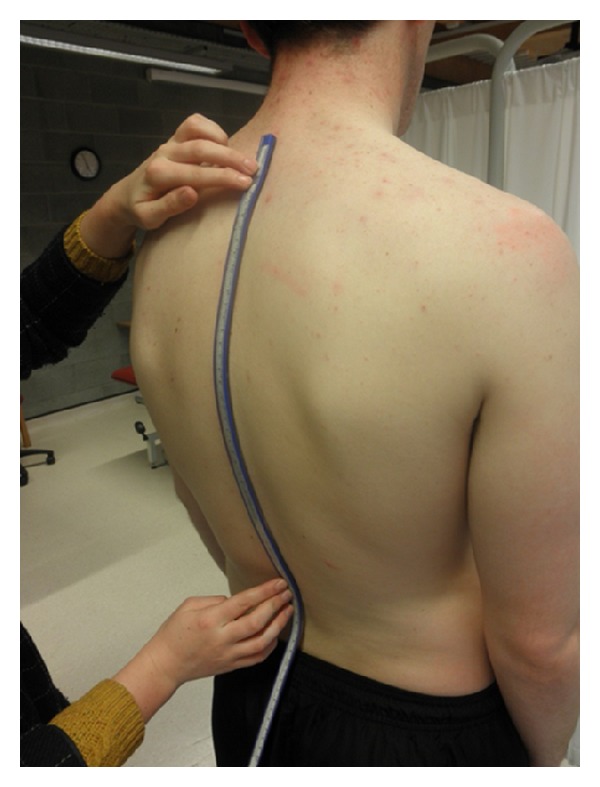
Application of flexicurve.

**Figure 2:**
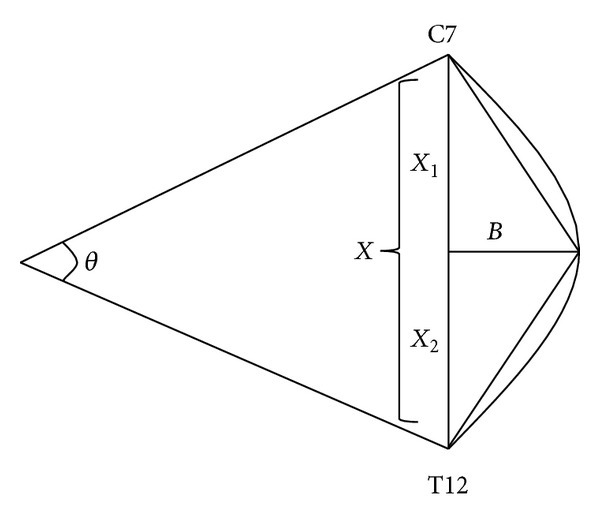
The flexicurve kyphosis index and angle are computed using measurements taken from the flexicurve tracing, represented here by the curve from C7 to T12. To calculate the flexicurve kyphosis index, the apex kyphosis height (*B*) is divided by the length of the entire thoracic curve (*X*) and then multiplied by 100 (*B*/*X* × 100). The flexicurve angle or theta (*θ*) is calculated using lines drawn perpendicular to the short sides of the triangle inscribed by the thoracic curve. Theta equals arc tan (*B*/*X*_1_) + arc tan (*B*/*X*_2_) [23].

The subject then assumed a standing position and was instructed to “adopt a comfortable position that felt natural to him/her” [29]. Standardized instructions reduced the possibility of the subject assuming different postures for the test and retest. The thoracic kyphosis was first measured using the flexicurve. As depicted in Figure 1, the tip of the flexicurve was placed over the C7 spinous process and the ruler was moulded to the contour of the thoracic spine. The flexicurve was carefully transferred to paper and the curve was outlined. The flexicurve was moulded to the spine 3 times, being flattened between each measurement. The kyphosis index was later calculated using the formula displayed in Figure 2. Calculations were undertaken in a separate session to ensure that rater 1 was blind to measures during the second testing session. The average of the 3 measurements of each subject was later used for analysis. Using geometric formulae, the flexicurve kyphosis angle was also calculated by the flexicurve tracing, as outlined in Figure 2.

**Figure 3:**
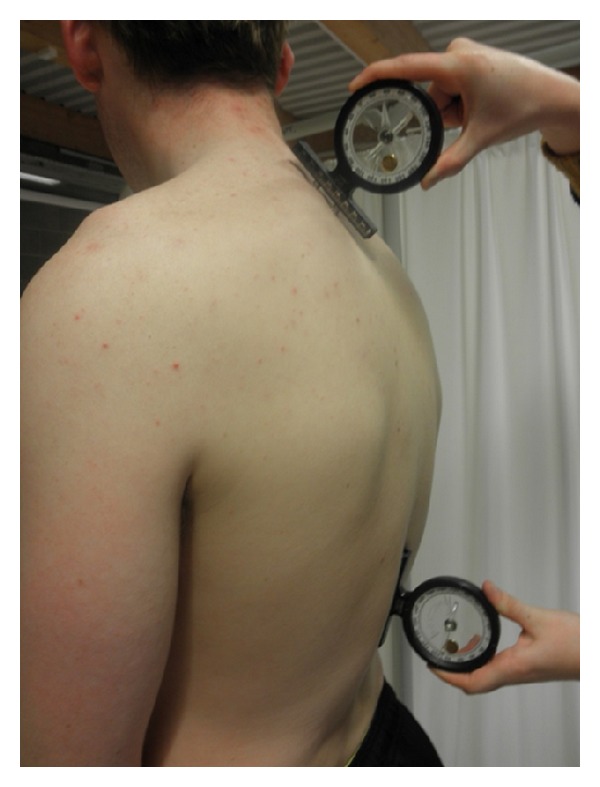
Application of the inclinometers.

Next the thoracic kyphosis was measured using two gravity-dependent inclinometers (Isomed, Inc., 975 SE Sandy Boulevard, Portland, OR, USA). As depicted in Figure 3, the feet of the inclinometers were placed over the spinous processes of T1/T2 and T12/L1. The readings were taken and recorded by a separate recorder to ensure blinding. The feet of both inclinometers were 2.5 cm apart, which remained constant for all subjects and testing sessions. Inclinometer measurements were performed 3 times in succession and an average was later used for analysis [28, 29].

After this initial testing session, the subject underwent separate tests with separate examiners, which consisted of scapular positioning measurements in standing and shoulder strength measurements in prone. Following these, the subject returned to rater 1 for retesting of thoracic kyphosis. In order to assess interrater reliability, 12 subjects were chosen at random. Immediately after completion of measurements with rater 1, rater 2 independently undertook the same protocol as rater 1. Spinal landmarks were repalpated for the second testing by rater 1 and again by rater 2. Rater 2 was not present for the measurements taken by rater 1, ensuring blinding between raters.

### 2.4. Statistical Analysis

Data was analyzed using SPSS software, version 11.0 for Windows (SPSS, Inc., Chicago, IL, USA). The mean, standard deviation, and range of thoracic kyphosis using the flexicurve index, flexicurve angle, and inclinometer were computed using descriptive statistics. Intra-rater and inter-rater reliability was determined by means of ICC, 95% confidence intervals, and standard error of measurement (SEM). ICC model 2 has been suggested to be best suited for generalizing the findings to clinicians with similar clinical experience [35]. Therefore, the ICC (2, 3) model for average measures was chosen. For the 12 participants in the inter-rater subset, the average of the three measures from the primary rater was compared with the single measure from the secondary rater in order to calculate inter-rater reliability. The following previously established categories for expressing levels of reliability were used: <0.40, poor reliability; 0.40 to 0.75, fair to good reliability; and >0.90, excellent reliability [36]. A linear regression was conducted to investigate the association between the flexicurve angle and inclinometer angle. Using linear regression, a formula was computed to approximate the flexicurve angle from the inclinometer angle. This takes the following form: flexicurve angle = (*β* coefficient × inclinometer angle) + intercept. Likewise, a formula was computed to approximate the inclinometer angle from the flexicurve angle. This takes the following form: inclinometer angle = (*β* coefficient × flexicurve angle) + intercept.

## 3. Results

**Figure 4:**
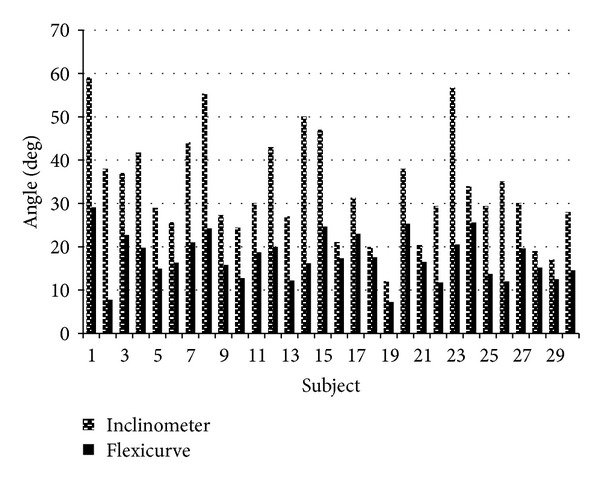
Bar chart displaying the inclinometer angle and corresponding flexicurve angle obtained for each subject by rater 1 on occasion 1. Evidently, the flexicurve angle is repeatedly less than the inclinometer angle.

Subject demographic data is presented in Table 1. The weekly swimming distance of included participants ranged from 6 km to 13 km. Sixteen out of thirty subjects had a history of shoulder pain which has prevented them from swimming for at least one week. Eight out of thirty swimmers currently have shoulder pain in at least one shoulder.  Table 2 displays the mean, standard deviation, and range of thoracic kyphosis values obtained from the flexicurve index, flexicurve angle, and the inclinometer.  Table 3 shows the intra-rater and inter-rater ICC and 95% confidence intervals. As a whole, interrater reliability of all methods was lower than intrarater reliability. Intrarater reliability was excellent and very similar for all methods, although the flexicurve index and flexicurve angle demonstrated slightly higher intra-rater reliability than the inclinometer. By contrast, the interrater reliability of the inclinometer was higher than that of the flexicurve index and flexicurve angle. The inclinometer showed excellent interrater reliability, while the flexicurve index and angle showed good reliability. The SEM result based on the ICC (2, 3) data for interrater reliability was 1° for the flexicurve angle, 2.2° for the inclinometer angle, and 0.4 for the flexicurve index. There was a poor association between the flexicurve angle and inclinometer angle (*R* ^2^ = 0.384).  Figure 4 displays a bar chart showing the differences between the flexicurve angle and inclinometer angle. These differences range from 2.4° to 36.2°. The mean difference between the two angles is 15.7°. The following equation was computed using linear regression to approximate the flexicurve angle from the inclinometer angle: flexicurve angle = (0.275 × inclinometer angle) + 8.478. To approximate the inclinometer angle from the flexicurve angle, the following equation should be used: inclinometer angle = (1.396 × flexicurve angle) + 8.694.

## 4. Discussion

The primary focus of this study was to compare the intrarater and interrater reliability of two clinical instruments for measuring thoracic kyphosis: the flexicurve and the manual inclinometer. The key findings indicate that in a sample of swimmers with and without shoulder pain the inclinometer demonstrated excellent intra-rater and inter-rater reliability. The flexicurve index and flexicurve angle both displayed identical reliability, with excellent intrarater reliability and good interrater reliability.

The secondary purpose of this study was to intercompare the inclinometer angle and the flexicurve angle. It is clear from Figure 4 that the thoracic kyphosis angles obtained by the flexicurve and the inclinometer have large inherent differences, with the flexicurve angle being much smaller than the inclinometer angle on every occasion. This trend was also revealed previously when Greendale et al. [23] compared the formulated flexicurve angle to Debrunner's kyphometer angle and the Cobb angle. As noted previously, the flexicurve angle is an inscribed angle, which by definition will be smaller than the circumscribed angles estimated using the Cobb or Debrunner methods [23]. Similar to these methods, the inclinometer provides a circumscribed angle which is evidently larger than the flexicurve angle. Conversion equations have been provided by this study, which may facilitate within-tester comparison of methods. Caution would be advised in the use of this conversion factor to nonswimming populations, until it is replicated in another population.

### 4.1. Intrarater Reliability

The excellent levels of intra-rater reliability (ICC = 0.94) of the flexicurve index reported in this study are in strong agreement with previous studies that used nonathletic populations. Teixeira and Carvalho [5] report ICCs of .87 for intra-rater reliability in a sample of 56 healthy participants of mean age of 66 years. Similarly, Yanagawa et al. [27] reported an ICC of .93 in 26 osteoporotic women of mean age of 67 years. The high intra-rater reliability (ICC = 0.92) of the inclinometer found in this study is also in agreement with previous studies. Greendale et al. [23] investigated intra-rater reliability in 45 subjects with and without shoulder pain. In the asymptomatic and symptomatic groups, Greendale et al. [23] demonstrated that ICC (2, 3) = 0.97. These results are in strong agreement with van Blommestein et al. [29] who also demonstrated excellent intra-rater reliability of the inclinometer [ICC (2, 3) = 0.96].

### 4.2. Intrarater Reliability

This study allows direct comparison between the inter-rater reliability of the flexicurve and the inclinometer as the participant sample is identical, the raters are identical, the landmarks palpated are the same for both instruments, and the time between rater 1 and rater 2 measurements is the same.

The level of interrater reliability (ICC = 0.86) of the flexicurve index reported in this study is lower than that reported in the past. Previously, two separate studies reported an ICC of 0.94 in healthy samples [5, 21]. In addition, Greendale et al. [23] report an inter-rater ICC of 0.96 in 166 elderly participants with hyperkyphosis. There are three sources of random error that may have influenced the reliability results obtained by this study: the equipment, the patient, and the clinician [37]. Due to the unstable nature of thoracic kyphosis [35], the kyphosis of the subjects may have changed between the measurements of rater 1 and rater 2, during which the subjects undertook strength and ROM measures. Alternatively, there may have been a discrepancy in the palpated landmarks of both raters. These factors, however, are questionable as the interrater reliability of the inclinometer remained high. A possible contributing factor to the lower interrater reliability of the flexicurve is the different levels of experience of each rater in using it. One rater had little prior clinical use with the flexicurve, while the other had years of clinical experience. Differences in the use of the flexicurve may have involved variation in amount of pressure applied with the instrument and in translating the flexicurve to paper. These challenges have previously been acknowledged [26, 38]. To the best of our knowledge, there is a noticeable absence of studies reporting inter-rater reliability of the manual inclinometer in the literature. The excellent levels of reliability observed in this study may facilitate the use of the inclinometer more widely in practice.

### 4.3. Clinical Significance

The use of a simple, quick, and reliable method for quantifying thoracic kyphosis is of value to clinicians, especially to physiotherapists working with large groups of swimmers. This study indicates that thoracic kyphosis can be measured reliably with a flexicurve or an inclinometer if the same clinician is to repeat the measurements. However, the inclinometer had higher interrater reliability than the flexicurve, which may favor its use in the swimming population. Furthermore, the inclinometer allows for instant interpretation of measurements compared to the flexicurve index and angle which require subsequent calculations. The values obtained from the spinal measurements described in this study, in both degrees and kyphosis index, could be used by clinicians to aid interpretation of values and to help to provide patient feedback. This is the first study to present the flexicurve angle as described by Greendale et al. [23] alongside the inclinometer angle. As reported previously, the flexicurve angle obtained by this method is on average approximately 15° less than the inclinometer angle [23]. The present study offers metrics that allow researchers and clinicians to scale the flexicurve angle to an approximate inclinometer angle in swimmers. The flexicurve angle (1°) produced a lower SEM value than the inclinometer angle (2.2°), which is more favorable clinically.

### 4.4. Limitation of Study

There are limitations to this study. The number of the subjects in the inter-rater subset was quite small. Future studies examining the inter-rater reliability of both instruments should incorporate a larger sample of participants. Although this study demonstrates high reliability for the inclinometer assessment tool, it does not demonstrate its validity as a measure of thoracic curvature. In order to establish validity, further research, comparing spinal angles obtained from an inclinometer with those obtained from radiographic investigations, the gold standard, is required.

## 5. Conclusion 

This study concludes that the inclinometer has excellent levels of intrarater and interrater reliability and clinicians can be confident of its reliability in a group of swimmers. Both the flexicurve index and angle have excellent intra-rater and good inter-rater reliability. Advantages associated with both the inclinometer and flexicurve for use at poolside are their ease of use, cost effectiveness, and portability. These positive characteristics combined with their high levels of reliability should encourage clinicians to use these tools to help guide treatment progression or monitor kyphosis levels. The flexicurve angle may be the most attractive of all 3 methods due to its lower SEM. However, caution must be used when interpreting flexicurve angles calculated by this method, in relation to the other methods. The comparison between the flexicurve angle and other methods is not valid, as it is repeatedly smaller than the Cobb angle [23] and the inclinometer angle.

## Figures and Tables

**Table 1:** Characteristics of subjects.

	Full sample (*n* = 30)	Interrater subset (*n* = 12)
Gender	Male = 18, Female = 12	Male = 5, Female = 7
Age (years)	45 (SD ± 16)	49 (SD ± 18)
Body height (cm)	172.8 (SD ± 7.9)	172 (SD ± 10)
Body mass (kg)	73.9 (SD ± 11.1)	74 (SD ± 15)
Years in a swim club	11 (SD ± 9)	10 (SD ± 10)
Average weekly swim distance (km)	9.9 (SD ± 14)	7 (SD ± 6)

**Table 2:** Descriptive statistics.

	Flexicurve index	Flexicurve angle	Inclinometer angle
Mean	7.7 (F = 7.59, M = 7.8)	17.6 (F = 16.2, M = 18.6)	33.3 (F = 30.6, M = 35.1)
Standard deviation	2.2 (F = 1.9, M = 2.4)	5.4 (F = 4.7, M = 5.7)	12.2 (F = 12.1, M = 13.2)
Minimum	3.1 (F = 5.43, M = 3.12)	7.3 (F = 7.8, M = 7.3)	12 (F = 17, M = 12)
Maximum	13 (F = 10.8, M = 13)	29.1 (F = 24.7, M = 29.1)	59 (F = 50, M = 59)

M: male, F: female.

**Table 3:** Intrarater and interrater reliability data.

	Intrarater reliability		Interrater reliability	
	ICC	95% CI	ICC	95% CI
Flexicurve index	0.94	0.88–0.97	0.86	0.51–0.96
Flexicurve angle	0.94	0.88–0.97	0.86	0.51–0.96
Inclinometer angle	0.92	0.84–0.96	0.9	0.68–0.97
